# Review Insights In Cardiac Tissue Engineering: Cells, Scaffolds, and Pharmacological Agents

**DOI:** 10.22037/IJPR.2021.114730.15012

**Published:** 2021

**Authors:** Safieh Boroumand, Azadeh Haeri, Niloofar Nazeri, Shahram Rabbani

**Affiliations:** a *Department of Tissue Engineering and Applied Cell Sciences, School of Advanced Technologies in Medicine, Shahid Beheshti University of Medical Sciences, Tehran, Iran. *; b *Department of Pharmaceutics and Nanotechnology, School of Pharmacy, Shahid Beheshti University of Medical Sciences, Tehran, Iran. *; c *Protein Technology Research Center, Shahid Beheshti University of Medical Sciences, Tehran, Iran. *; d *Research Institute for Prevention of Non-Communicable Diseases, Qazvin University of Medical Sciences, Qazvin, Iran. *; e *Research Center for Advanced Technologies in Cardiovascular Medicine, Cardiovascular Diseases Research Institute, Tehran Heart Center, Tehran University of Medical Sciences, Tehran, Iran.*

**Keywords:** Stem cells, Biomaterials, Angiogenesis, Cardiac tissue engineering, Regeneration

## Abstract

Heart failure (HF) is one of the most important cardiovascular diseases (CVD), causing many die every year. Cardiac tissue engineering is a multidisciplinary field for creating functional tissues to improve the cardiac function of the damaged heart and get hope for end-stage patients. Recent works have focused on creating engineered cardiac tissue *ex-vivo*. Simultaneously, new approaches are used to study ways of induction of regeneration in the damaged heart after injury. The heart as a complex physiological pump consists of many cells such as cardiomyocytes (80–90% of the heart volume). These cardiomyocytes are elongated, aligned, and have beating properties. To create the heart muscle, which should be functional, soft and elastic scaffolds are required to resemble the native heart tissue. These mechanical characteristics are not compatible with all materials and should be well selected. Some scaffolds promote the viability and differentiation of stem cells. Each material has advantages and disadvantages with relevant influence behavior for cells. In this review, we present an overview of the general approaches developed to generate functional cardiac tissues, discussing the different cell sources, biomaterials, pharmacological agents, and engineering strategies in this manner. Moreover, we discuss the main challenges in cardiac tissue engineering that cause difficulties to construct heart muscle. We trust that researchers interested in developing cardiac tissue engineering will find the information reviewed here useful. Furthermore, we think that providing a unified framework will further the development of human engineered cardiac tissue constructs.

## Introduction

Myocardial infarction (MI) is the leading mortality cause worldwide. Limited regenerative potential of cardiac tissue following ischemia leads to scar formation in the infarcted region. Besides, dysfunction of electrical signal propagation disturbs the synchronization of cardiac contraction, which leads to heart failure (1). 

Because of the inadequacy of existing therapies for healing of damaged hearts and the shortage of donations for heart, different strategies have emerged in myocardial regeneration (2). Cardiomyoplasty is wrapping the heart with muscles which can be expressed as a sort of heart patch applied to about 2000 people in clinical trials but failed to show reliable results (3). Another promising therapeutic item that researchers have considered to regenerate infarcted myocardia is cell-based therapy, however, the challenge of cell engraftment must be overcome to increase the efficacy of this approach (4-6). A discussed reason for poor therapeutic results of transplanted cells into the infarcted area can be the low rate of engraftment and high mortality rate of transplanted cells due to mechanical cell leakage (7, 8). 3D scaffolds can be designed by tissue engineering to mimic the extracellular matrix (ECM) to enhance cell survival and engraftment. Besides, an engineered scaffold can be used as a platform for different growth factors to impact engrafted cells positively (9). 

In the late 1990s, researchers began to engineer cardiac patches (10-12), and experiments continued to animal studies (13) and clinical trial studies (14-16). Researchers examined different approaches to evaluate a proper 3D scaffold for myocardial regeneration. Methods that can be mentioned for the construction of polymeric 3D scaffolds are electrospinning, freeze-drying, thermally induced phase separation, 3D bioprinting, solvent casting/particulate leaching, gas foaming, photolithography, and laser ablation (9). 

Studies on animal models showed that combining cell therapy with tissue engineering techniques to create cell sheets and patches can increase stem cell survival and boost therapeutic action (4). Therefore, tissue engineering has been considered as a potential approach for cardiac regeneration after MI. Current research in this field is, to a large extent, aimed towards the development of functional heart tissue for application in cell-based regenerative therapies. In particular, challenges in cardiac tissue engineering and difficulties in cardiac regeneration have been discussed in this article in the next section. Engineered cardiac tissues remain immature and still differ from the adult human heart. One of the significant challenges in cardiac tissue engineering is the organization of cells into a functional tissue suitable for therapeutic use. In this review, we describe the main approaches undertaken toward the development of tissue-engineered constructs, and discuss the current state and future directions.

**Challenges in heart regeneration**

The human heart pumps blood through the body about 80 times per minute, 2 to 3 billion times over a lifespan. This indicates that the heart has a complex nature. Heart failure with reduced ejection fraction is most often caused by coronary artery disease, arterial hypertension, or cardiomyopathies. Even though the causes are diverse, heart failure results from primary cardiomyocyte dysfunction (e.g., genetic cardiomyopathies) or a loss of cardiomyocytes (e.g., after myocardial infarction). Current pharmacological treatment reduces mortality and morbidity but does not generate new cardiomyocytes (17).

Unlike skeletal muscle, the heart lacks enough resident stem cell population, and cardiomyocytes cannot be entered the cell cycle after birth (18). During a short postnatal period, myocardial injury causes an increase in cardiomyocyte proliferation, and the heart can regenerate. This process contains the formation and integration of new myocytes and revascularization. In the adult heart, cardiomyocyte renewal is minimal, and cardiac injury leads to fibrosis and scar tissue formation (19). 

The adult mammalian heart cannot stand injury, such as ischemia. It is well known now that irreversible ischemic myocardial cell injury develops the number of compromised cells in two to three hours. In response to ischemia, the cardiac repair mechanism in the adult heart represents an impaired healing response, leading to cardiomyocyte hypertrophy with fibrosis, which results in remodeling of the heart and failure. Currently, the best treatment for cardiac failure is heart transplantation, which is influenced by donor supply and compatibility (20).

Several cardioprotective strategies have been utilized to protect the heart during ischemia. Hypothermia (32–35 °C) activates signaling mechanisms either the extracellular signal-regulated kinases (ERK1/2) and/or the Akt/phosphoinositide 3-kinase/mammalian target of rapamycin pathways and reduce myocardial infarct size (21). This process includes three mechanisms: ischemic preconditioning, ischemic postconditioning, and remote ischemic preconditioning (22). Several potential signaling and molecular pathways have been proposed as intracellular mechanisms for cardioprotection. Researchers have recently detected the hypothesis that these processes are affecting mitochondrial function. Mitochondrial damage affects cardiomyocyte function and disrupts oxidative phosphorylation, Ca (2+) dyshomeostasis, and increases oxidative stress. The process is started when the high-conductance channel located at the contact sites between the inner and outer mitochondrial membranes, called Mitochondrial Permeability Transition Pore (MPTP), opens for longer terms and breaks up the inner mitochondrial membrane potential. The activation of PI3K, phosphoinositide-dependent kinase, Akt, and ERK (RISK Pathway) by preconditioning targets the glycogen synthase kinase 3β (GSK 3β) and inhibit the opening of MPTP (23). NO is an essential molecule in preconditioning through the increase of cGMP formation and activation of PKG. NO targets mitochondrial proteins through the nitrosination or nitrosylation process. Nitrosation of mitochondrial respiratory chain complex I and mitochondrial connexin 43 (mtCx43) are crucial in the cardioprotective effects mediated by pre and post-conditioning (24). The ATP-dependent potassium channel (K_ATP_) at the inner mitochondrial membrane is involved in the protective influence exerted by all conditioning types. Although mechanisms are not completely clear, the mitochondrial K_ATP_ channel, upon its activation (following mitochondrial translocation of PKC), releases reactive oxygen species (ROS), which, in turn, activates the PKCε in a positive feedback loop(25). 

Pharmacological agents can reduce morbidity and mortality associated with myocardial infarction. Current international guidelines relieve the cardioprotective benefits of beta-blockers in myocardial infarction, and reducing the force of contraction and heart rate, leads to decreased myocardial oxygen demand(26). This mechanism has been proposed as the basis of the cardioprotective effects of beta-blockers after large myocardial infarction (27). 

Recently, cell therapy has been performed with cell replacement to the injured myocardium with functional cardiomyocytes. The adult cell transplantation approach is widely used because of its ease of use and good safe manner. To date, cell therapy has been effective in reducing cardiomyocyte apoptosis and tissue fibrosis while promoting angiogenesis and immunomodulation of inflammation via modulatory stimulation of the microenvironment (28). It is known that trophic molecules secreted by the transplanted cells are more critical than the differentiation potential of the cells. In this scenario, cell-secreted factors can be developed a defective cardiac repair process by supporting the survival of resident cardiomyocytes, activating therapeutic angiogenesis, and modulation of inflammation, which leads to less fibrosis (29). 

**Materials for scaffold construction**

**Figure 1:**
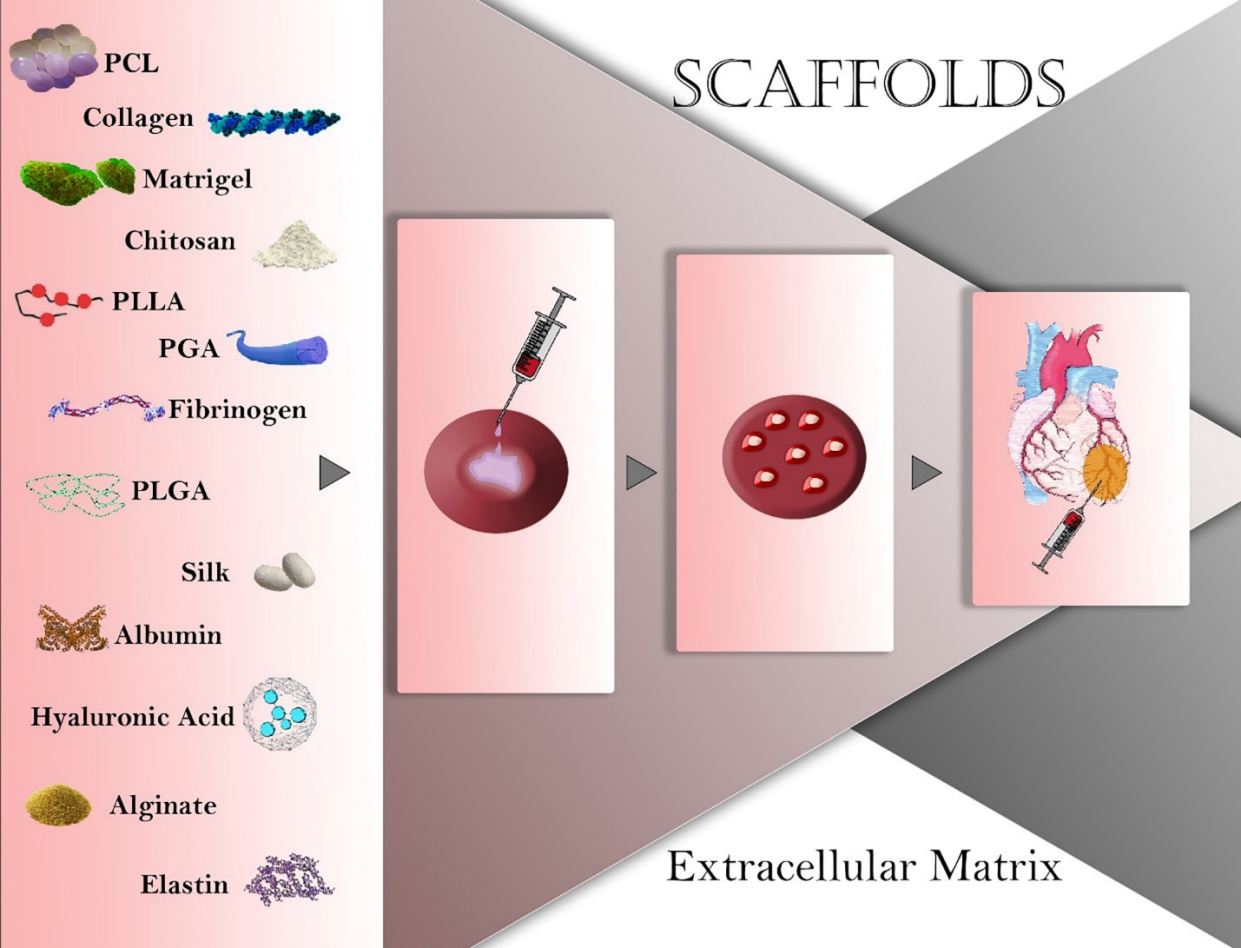
Natural and synthetic materials used in cardiac tissue engineering. PCL: polycaprolactone; PLLA: polylactide; PGA: Polyglycolide; PLGA: poly (lactic-co-glycolic acid).

Components and architecture of myocardium ECM have a specific pattern for researchers to engineer a cardiac patch. Collagen (30, 31) and fibrin (32) as the main components of ECM have been used by different studies for the construction of 3D scaffolds to enhance infarcted heart-healing. Other common natural materials for cardiac tissue engineering are chitosan, gelatin, elastin, and silk (Figure 1). However, to upgrade the desired scaffold for cardiac tissue engineering, most researchers used a composite or a mixture of different natural and synthetic polymers to cover their undesired features (9) (Table 1). 

*Natural materials*

Natural polymers like collagen, chitosan, fibrinogen, silk, albumin, hyaluronic acid, alginate, and elastin have proper biochemical features for cell attachment and proliferation (33). Also, natural polymers are non-toxic with low immunogenicity. However, there are some drawbacks such as the crosslinking step for the insolubility of natural polymers during the fabrication process might change their 3D structure and porosity (34). Besides, cross-linker agents such as hexamethylenetetramine and glutaraldehyde can affect the biocompatibility of natural polymers (35). Collagen, fibrinogen, chitosan, elastin, gelatin, and silk are common natural polymers used for myocardial tissue engineering (9).

*Collagen*

An essential component in myocardial connective stroma is collagen, especially collagen type I and III, which are widely considered for cardiovascular engineering (9). Norahan fabricated cardiac collagen patches via freeze-drying method with incorporated graphene oxide for its electroactive properties, the patches showed angiogenesis property that is favorable for cardiac tissue engineering (36). Other experiments were carried out on collagen as a promising factor for cardiac tissue engineering (37-41).

*Decellularized myocardium*

The application of decellularization in cardiac tissue engineering has rapidly progressed in the past 10 years. In 2008, Ott *et al. *reported the development of an acellular rat whole heart via coronary perfusion(42). The decellularized rat heart preserved the complex ECM composition and retained intact chamber geometry and perfusable vascular architecture. In the subsequent years, whole-organ decellularization approaches have been extended to the larger hearts from porcine and human origin to realize human-size functional cardiac grafts. For example, Wainwright *et al. *conducted the first study to generate an acellular porcine heart. The decellularized porcine heart preserved ECM composition (e.g. collagen, elastin, and glycosaminoglycans), retained mechanical integrity, and supported cardiac cells *in-vitro* (43). Compared to the porcine heart, the decellularization method has been applied to a limited number of the human heart to obtain dECM scaffold. Sanchez *et al. *produced the first acellular human heart scaffold. After decellularization, the human heart preserved the three-dimensional (3D) architecture, chamber geometry, vascularity, and mechanical anisotropy. When reseeded with parenchymal and vascular cells, the human heart promoted cardiocyte gene expression and electrical coupling (44). Decellularization has also been exploited to derive cardiac decellularized ECM (dECM) slices. Various groups have fabricated cardiac dECM slices through decellularization using native cardiac tissues from multiple species including rats, mice, pigs, and humans. Moreover, the dECM slices have been explored for many *in-vitro *and *in-vivo* studies to investigate the dECM properties and cell-matrix interaction (e.g. cell adhesion, proliferation, and differentiation) using various cell types including mesenchymal stem cells, embryonic stem cells, and induced pluripotent stem cells (45-49). dECM derived from myocardium tissue has gained significant attention for cardiac repair and regeneration. The use of decellularization and recellularization strategies have enabled to produce cardiac dECM scaffolds that mimic tissue properties (e.g. ECM architecture, biochemical cues, and mechanical integrity) similar to the native heart. Because of these attractive advantages, the cardiac dECM scaffold can provide microenvironment and biological signals for the damaged heart that could promote tissue reconstruction upon implantation. Long-term investigation after implantation needs to be performed before commercialization and clinical trial. Nevertheless, it is undoubted that the decellularized heart ECM scaffolds will be attractive candidates for clinical trial once they can be further characterized.

*Chitosan*

Chitosan is another natural polymer that is used for cardiac tissue engineering because of its biodegradability, biocompatibility, and non-toxicity (9). The most common method to obtain chitosan is alkaline deacetylation of chitin (50). The application of chitosan with polypyrrole modification on its surface to construct a conductive cardiac patch has been studied. Their results revealed improved cardiac pumping in the rat MI model (1). In another study, titanium dioxide (Tio_2_) nanoparticles were added to PEGylated chitosan (covalent conjugation with polyethylene glycol) hydrogels to improve their biological and physicochemical properties. The results of the *in-vitro* study showed excellent cell-hydrogel matrix interactions with cardiomyocytes cells (51). 

*Fibrinogen*

Fibrinogen is a glycoprotein present in the human blood plasma and can promote cellular interaction and scaffold remodeling (9, 52, 53). Poly (glycerol sebacate)/fibrinogen nanofibrous mat with a core-shell structure containing vascular endothelial growth factor (VEGF) showed the ability to differentiate human bone marrow mesenchymal stem cells (MSCs) into cardiac cells (54). Monomers of fibrinogens convert to fibrin via thrombin exposure, and this approach can be used to deliver a mixture of cells and fibrinogen to the infarcted regions of the heart (55, 56).

*Silk*

Silk as a natural protein has been used in tissue engineering of cardiac patches due to its biocompatibility and proper mechanical properties (9, 57-61). Stoppel *et al. *developed a silk-based scaffold that included cardiac tissue-derived extracellular matrix (cECM). *In-vitro *results suggested that silk-based scaffolds can improve the functional phenotype of HL-1 atrial cardiomyocytes and human embryonic stem cells derived cardiomyocytes. Furthermore, *in-vivo* experiment revealed that cells infiltrate significantly through aligned silk scaffold with promoted vascularization (62). 

*Albumin*

Albumin is the most abundant plasma protein which has been considered for cardiac tissue engineering. Albumin is a biocompatible and biodegradable source for tissue engineering, which can be obtained from patients` serum (63-66). Improved elasticity of electrospun albumin compared to synthetic electrospun polycaprolactone (PCL) fibers was reported by Fleischer *et al. *(65). This group continued their studies over the capability of albumin for cardiac patches and found that electrical properties of albumin-based scaffolds improved with gold electrodes (66) and suture-free patches of albumin designed with gold nanorods (63). 

*Hyaluronic acid (HA)*

Hyaluronic acid (HA) is another natural material that has been used as a hydrogel for cardiac repair. This unbranched glycosaminoglycan polymer is distributed in the extracellular matrix. Its important biological activities for tissue engineering are angiogenesis potential and suppressing fibrous tissue formation. Injection of HA hydrogels into the infarcted area of the rat can facilitate the functional recovery of cardiac tissue (67). Another study reported that HA can increase the contraction force of collagen *in-situ* cross-linking hydrogels (68). A research team transplanted a 3D-printed scaffold of hyaluronic acid/gelatin with human cardiac-derived progenitor cells in a mouse model of MI. Their results showed a reduction in adverse remodeling and promotion in cardiac performance (69).

*Alginate*

Alginate is a polysaccharide derived from algae with gelling properties, low toxicity, and high biocompatibility. A study reported that a 3D scaffold of alginate and chitosan could increase ejection fraction, improve neovascularization, and attenuate fibrosis (70).

*Elastin*

A few studies reported the use of elastin for cardiac patch fabrication. This natural polymer, a protein in ECM, with an elastic nature and low mechanical response, can be used for the cardiac patch in combination with other polymers like PCL or collagen to improve its mechanical properties (71-73).

*Synthetic materials*

Synthetic polymers are human-made polymers and cannot be found in nature. These polymers and their mechanical properties are highly controllable (74). 

*Polycaprolactone*

Polycaprolactone (PCL) is abundantly used for myocardial tissue engineering by different researchers. Shine *et al. *constructed cardiac contractile grafts with culturing of cardiomyocytes on nanofibrous PCL scaffolds without any extra modification (75). Since then, other studies have used hybrid PCL scaffolds with natural or other synthetic polymers or used different modifications to improve PCL scaffolds properties for cardiac tissue engineering. 

 The research used PCL in combination with natural polymers of collagen, elastin, and gelatin to avoid the crosslinking process of natural polymers. However, the tensile strength of gelatin/PCL scaffolds was higher than collagen/elastin/PCL. Proper cell attachment was observed on the surfaces of both scaffolds, but cell migration was reported predominantly in the gelatin/PCL hybrid scaffold (76). Another study used collagen coating on aligned PCL nanofibers, and it could improve the survival of cardiac cells compared with non-coated and random PCL nanofibers (77).

According to the hydrophobic nature of PCL, surface functionalization of PCL can improve its cell-matrix interactions. Due to this fact, Guex *et al. *used a radio-frequency (RF) plasma process to functionalize electrospun PCL patches (78). In another study, oligomer hydrogel bisphenol A ethoxylated dimethacrylate (BPAEDMA) was used in combination with PCL to reduce the stiffness and hydrophobicity of PCL. This compound also promotes cell adhesion and proliferation (79). Recently, poly (glycerol sebacate) (PGS) has emerged in the biomedical application, and a combination of PGS and PCL showed improved cell attachment and growth of myogenic and vasculogenic cell lines compared to PCL alone (80). Other hybrid PCL scaffolds were constructed with polyhydroxyalkanoate/PCL, silk fibroin/PCL, and polyurethane/PCL, which improved the regenerative potential of PCL for cardiac tissue engineering (81-83).

PCL-based scaffolds’ conductivity increased with applying nanoparticles, conductive polymers, carbon nanotubes, graphene, and piezoelectric microfibers that positively affected cardiogenic differentiation toward typical cardiac phenotype (84-87). 

*Poly (L-lactic acid)*

Poly (L-lactic acid) (PLA) as a biodegradable biopolymer is another synthetic polymer that is used for cardiac tissue engineering (88, 89). Chung *et al. *fabricated PLA based electrospun mat for delivering VEGF and cardiac stem cells (CSCs) for the acute infarcted heart. Results demonstrated that angiogenesis and cardiomyogenesis significantly improved by using these scaffolds (90). In another recent study, PLA was used to deliver granulocyte colony-stimulating factor (GCSF) to the region of the infarcted myocardium. This study revealed the angiogenesis potential of PLA/GCSF sheet, which could prevent ventricular dilatation with improving cardiac performance (91). Liu’s research team fabricated a composite of PLA/collagen with the potential of myocardial regeneration and demonstrated that aligned scaffolds could support cardiomyocyte viability more than random scaffolds (92). A composite of PLA and polyaniline was designed by Wang to improve the conductivity of PLA scaffold. Primary cardiomyocytes, with improved cell-cell interaction and maturation, spontaneously beat in this conductive sheet (93). Bertuoli *et al. *fabricated an electrospun core-shell scaffold composed of PLA and polyethylene glycol (PEG) with polyaniline. Even though dodecylbenzene sulfonic acid doped polyaniline had high cytotoxicity, but PLA/ polyaniline fibers showed a reduction in cytotoxicity because of polyaniline interactions with PLA. Incorporating PEG increased the conductivity properties of PLA/ polyaniline and its cytotoxicity related to the rapid dissolution of PEG in a culture medium, which led to increasing polyaniline release (89).

*Poly glycolic acid*

Poly glycolic acid (PGA) is a degradable polymer that is approved for biomedical applications. This semi-crystalline polyester is biocompatible, nontoxic, and hydrophilic making it interesting for tissue engineering. Also, its favorable mechanical properties and hydrophilicity are beneficial for tissue engineering when applied to a blend of other synthetic or natural materials (94, 95). A favorable ratio of electrospun PCL and PGA for myocardial tissue engineering was reported by Aghdam *et al. *It was observed that adding PGA to PCL increased its degradation rateand better cell adhesion and growth (94). Another study demonstrated that the presence of a PGA nanofibrous sheet in collagen could improve strength and cardiac stem cell attachment more than composite nanofibers of collagen and PGA (95).

*Poly (DL-lactide-co-glycolide)*

Poly (DL-lactide-co-glycolide) (PLGA) is a biocompatible and biodegradable polymer that can be modulated by varying PLA and PGA content and used for tissue engineering (96, 97). Furthermore, PLGA nanofibers can be loaded with drugs and used in drug delivery with appropriate drug release (98, 99). Cristallini *et al. *evaluated a cardiac patch with a blend of PLGA and gelatin to mimic the anisotropic structure and mechanical properties of the myocardium. Promoted adhesion and elongation of human mesenchymal stem cells observed by PLGA/gelatin scaffolds (98, 100). In another *in-vitro* study, human inducible pluripotent stem cell-derived cardiomyocytes were cultured on aligned PLGA scaffolds as a cardiac patch and expressed α-actinin, troponin-T, and connexin-43 similar to natural cardiac tissue structure (101). In another study, cardiac patch made of PLGA electrospun nanofibers were bio-functionalized with adhesive peptides, YIGSR (derived from fibronectin), and RGD (derived from laminin). This study demonstrated that aligned YIGSR-incorporated PLGA electrospun nanofibers can improve the contractile property of cardiomyocytes (102). 

**Required characteristics for tissue-engineered cardiac patches**

In myocardial tissue engineering, some parameters are necessary to be considered for mimicking natural tissue. The composition of materials, mechanical properties, surface characteristics, degradation rate, biocompatibility, and cell seeding condition are crucial parameters for myocardial tissue engineering. The importance of scaffold topography and its electrical conductivity has been proved in different studies (9) discussed here.

*Degradation*

Regeneration of the heart after MI lasts about 6-8 weeks, so it seems that the appropriate degradation time for a cardiac patch must be about two months. Because prolonged degradation time can induce chronic inflammatory responses and lead to fibrous capsule formation, it is necessary to design a cardiac patch with a proper degradation rate (83). Hashizume *et al. *evaluated the efficacy of cardiac patches with different degradation rates. In this study, three kinds of biodegradable polyurethane with fast (poly (ester urethane) urea: PEUU), moderate (poly (ester carbonate urethane) urea: PECUU), and slow (carbonate urethane urea: PCUU) degradation rates were examined in the rat model of MI. Evaluation for 16 weeks revealed that the presence of PECUU and PCUU in the implantation site could increase the active period of M2 macrophages. In this study, the functional and histological assessments revealed that cardiac patches with a moderate degradation rate led to the formation of a desirable extracellular matrix with markers of positive remodeling (103).

*Topography*

Applications of nano- and microstructure for myocardial tissue engineering were investigated in different studies. To mimic natural myocardial tissue structure, engineered scaffolds with an aligned fibrous anisotropic structure can improve the organization and proliferation of seeded cardiac cells (104-106). Ghosh evaluated the effect of scaffold structure on cardiomyogenesis of human MSCs in 3D scaffolds of PCL nanofibers and 2D PCL films. Expression of cardiac markers was elevated in 3D nanofibers, which were higher for aligned nanofibers instead of random ones. Also these cardiac markers were significantly elevated in 3D nanofibers in comparison to 2D films (107). Fleischer evaluated the behavior of cardiac cells on PCL-based spring-like and straight scaffolds. This study demonstrated that cardiac cells cultured on 3D thick spring-like fiber scaffolds exhibited improved function, including higher beating rate, lower excitation threshold, and stronger contraction compared to straight fiber scaffolds (108). 

*Mechanical properties*

Tissue-engineered myocardium should mimic the mechanical properties of the heart to promote the contractive characteristics of growing cells. It has been reported that the stiffness of the left ventricle is between 10-20 kPa (beginning of diastole) and 200-400 kPa (at the end of diastole) (83). However, this amount is 30-50 kPa for ischemic tissue associated with fibrosis (9). Based on different studies over the appropriate Young’s modulus for cardiac cells, the best range is between 10 kPa and 1 MPa (9). The elastomeric polymers can better imitate a native heart than stiffer biomaterials and promote cardiomyocyte’s growth and beating rate (83). 

*Conductivity*

The transportation of electric signals through myocardium tissue makes it possible for cardiac cells to beat. It is known that electrical stimuli can control the attachment, proliferation, and differentiation of cells by controlling the surface charge (9, 109, 110). Cardiac differentiation evaluated under applying exogenous electrical stimulation for different kinds of cells such as adipose-derived stem cells (111), MSCs (112-114), hESC (115, 116), fibroblast (117), iPSCs (110), and hCPCs (118, 119).

Different approaches are used by different researchers to mimic the electrical features of cardiomyocytes. Applying conductive nanomaterials and polymers in the construction of scaffolds can promote synchronous contractions of cardiac cells (120-123).

Carbon nanotubes (112, 124 and 125) and gold nanoparticles (84, 120 and 122) are nanomaterials commonly used to make conductive scaffolds for the cardiac patch. Incorporating carbon nanotubes into photo-cross-linkable gelatin methacrylate hydrogel improved cell adhesion and organization with resistance to cytotoxic compounds (125). In another study, adding the gold nanoparticles to injectable laponite-ECM hydrogels led to the developed expression of cardiac-specific markers like cardiac troponin I (cTnl) and connexin 43 (Cx43) (120).

Graphene is another material that can be used for the conductivity of a cardiac patch (114, 126). Stone used graphene oxide to create a conductive cardiac patch along with polyester amide (PEA) and PEA-chitosan fibrous scaffolds. A combination of PEA-chitosan and graphene oxide supports cardiac differentiation without cytotoxicity (114).

Conductive polymers like polypyrrole (85), polyaniline (89), poly (thiophene- 3-acetic acid) (PTAA) (127) have been used by different researchers for constructing conductive cardiac patches. Besides, other kinds of conductive polymers such as polythiophenes and poly (para-phenylene vinylene) have been reported to construct a conductive cardiac patch (123). 

**Nano-structures**

Materials and cells for myocardial tissue engineering are not the only crucial items for mimicking the physiological relevance of myocardial tissue. The ECM of the myocardial tissue has a well-organized and anisotropic architecture and conductive purkinje fibers that provide signaling cues between cardiac cells. Many approaches adopted micro and nano-scale technologies for a native-like cell biomaterial interaction in myocardial tissue engineering (128). 

Conductive nanomaterials like gold nanoparticles can be used to design an electric field stimulated culture condition to guide stem cell differentiation (129). An electrical signal that passes through a conductive scaffold can improve a tissue-engineered myocardium (109). Graphene and carbon nanotubes are organic nanomaterials which mostly used for constructing a conductive scaffold (128). Also, nanomaterials, like nanofibers can significantly enhance the retention of applied stem cells into the site of injection, leading to improved efficacy of cell therapy (130)

**Applied cell sources for myocardial tissue engineering**

**Figure 2:**
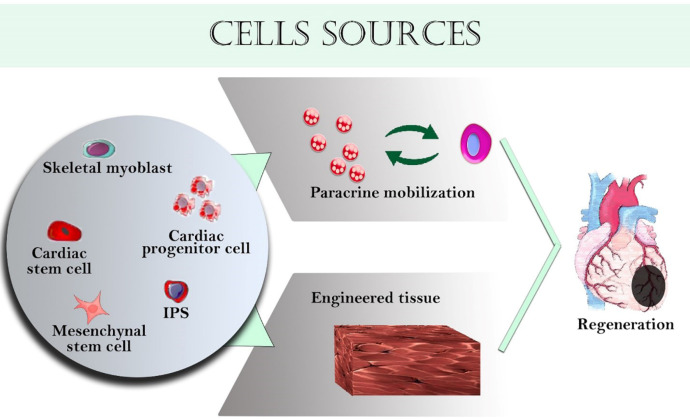
Various cells used in cardiac tissue engineering

The most common administration methods of cells in clinical trials are direct intra-myocardial injection and intravascular injection. However, the rate of survived cells at the site of administration is not efficient for effective treatment. In preclinical studies, it has been observed that cell retention in the site of administration can be improved with tissue-engineered scaffolds (2). Many cell sources have been used for cardiac tissue engineering (Figure 2), and each of them has advantages and disadvantages (Table 2). 

*Embryonic stem cells*

Embryonic stem cells (ESCs) can be collected from inner mass cells of blastocysts and can differentiate into cell types of all germ layers (131). Menasche used cardio-instructive cues to differentiate ESCs to cardiac progenitor cells and embedded them in a fibrin patch. Cardiac function improved via implantation of cells embedded patches on the infarcted area of a patient with severe heart failure (14). Before this clinical study, the efficacy of human embryonic stem cells embedded in a fibrin scaffold for the improvement of cardiac function has been proved by Bellamy *in-vivo* study (132).

Ke used a biodegradable PGA scaffold to seed ESCs and grafted it to the myocardial infarcted site of mice. This research revealed that ESCs could improve myocardial function; however, their findings showed that improvement in cardiac function via ESCs seeded patches was more than the injection of ESCs into the site of MI, without cell engraftment within scaffold (133). In another study, temperature-responsive chitosan hydrogel combined with ESCs was used for increasing cell retention in the site of MI in the rat model. Engraftment of ESCs into hydrogel significantly increased cardiac function compared to the administration of ESCs without scaffold (134). 

It is well established that mammalian ESCs can form complex teratomas when engrafted into a host, a characteristic of these cells resulting from their pluripotency (135). Teratomas caused by transplanted ESCs include highly organized and differentiated cell types representative of all three germ layers(136). The cell types identified using cell-specific markers include keratinized cells, hair follicles, muscle cells, cardiomyocytes, epithelial cells, neural ganglia, and pigmented cells (135). Teratoma formation is a significant potential limitation in the therapeutic use of ES cells.

*Induced pluripotent stem cells*

Induced pluripotent stem cells (iPSCs) are widely used for regenerative medicine and can be obtained from the patient’s somatic cells with overexpression of Oct4, Sox2, Klf4, and Myc (2). IPSCs are similar to ESCs and have been used to regenerate the heart in many animal studies, even though the concern about tumorigenesis potential of iPSCs should be considered (137, 138). Different studies revealed that iPSCs can promote cardiac function (139); however, some studies reported that iPSCs could be tumorigenic (138, 140). 

*Challenges of hPSCs in the treatment of myocardial damage*

It has been known that hESCs have low expression of major histocompatibility complex I and lack of major histocompatibility complex II antigens and CD80 and CD86. The expression of the above molecules in hiPSCs is the same as those in hESCs. Thus, hPSCs may possess immune responses (141). However, immunogenicity may be presented after transplantation. Immunosuppressive drugs can suppress the immune system, but optimal doses and combinations of different drugs are still far from optimization(142). HiPSCs can be differentiated into the recipient’s cells and tissues, but they may have limited therapeutic potential if they are reprogrammed from genetic mutation diseases. Currently, it is hard to provide evidence for the superiority of the efficacy of either hPSC-CMs or CPCs (143). Although many protocols have been used to induce hPSCs into other cells, there are no completely accepted standards to evaluate the quality of hPSC-derived cardiac cells especially hiPSC-derived cardiovascular cells, because the reprogramming process may change the genetic stability and may compromise therapeutic efficacy. It has been shown that hiPSC-CMs can induce arrhythmia such as abnormal impulse formation, conduction abnormalities, or disease phenotypes, for example, long QT syndrome and cardiomyopathies(144). 

Another issue is the immature phenotype of hiPSC-CMs, represented by structural disorganization or electrophysiological parameters (145, 146). This immaturity may induce action potential changes and display distinct gene expression profiles associated with multiple ion channel variants. Immature phenotype, is the disadvantage of hiPSC-CMs, and has led to the development of maturation protocols (147). 

IPSC-CMs can improve left ventricular function, infarct area, myocardial wall stress, myocardial hypertrophy, and reduce apoptosis of myocardial cells in the host tissue, but the integration between the graft and the host myocardium should be improved (148, 149). All of these issues make limitations of the use of these cells in clinical studies. 

*Cardiac stem cells*

Cardiac stem cells (CSCs) have the potential of self-renewing and can differentiate into different cell types, including vascular smooth muscle cells, cardiomyocytes, and endothelial cells (137). 

CSCs are multipotent, self-renewing, and can form cardiomyocytes, smooth muscle cells, and endothelial cells. In the adult heart, most of the CSCs are reside in the atrium and the ventricular apex, at a very low density (1 cell per every 10,000 myocytes). It is proposed that cell turnover in the mammalian heart muscle occurs at a very low rate, which may contribute to its structural maintenance (150). It is normally insufficient to heal the heart after injury, but conditions or drugs may be identified to stimulate the cells to renew. These cells are predicated to rare mitotic cardiomyocytes or on the existence of progenitor and stem cells in an adult cardiac niche (151). The key to understanding the fate of proliferating cells in the adult heart may be found during its development when active cell division is supported in dynamic cardiac microenvironments. CSCs can be proliferated over long-term culture and maintained undifferentiated and self-renewing without reducing growth or abnormal karyotype (152). Preclinical studies showed that CSCs regenerated the hearts of rats and mice post-infarction via the formation of new cardiomyocytes and vasculature, and protected the existing cardiomyocytes from apoptosis through the secretion of insulin like growth factor (153). 

The first experiment on the feasibility of using cardiac cells for myocardial regeneration was reported by Soonpaa in the early 1990s. Further *in-vitro *and *in-vivo* studies were continued for the regenerative potential of cardiac cells for the infarcted heart (2). Despite low retention of CSCs after intra-myocardial administration post-MI, CSCs significantly improved left ventricular function during 35 days (154). Different studies were performed to improve cell retention, and researchers found a significant improvement in myocardial capillary density by vascularized cardiac patch and CSCs (155). In another study by Zhang, mesenchymal stem cells derived exosomes used to enhance myocardial regeneration via stimulation of proliferation, migration, and angiogenesis of CSCs. *In-vivo* study showed that CSCs pretreated with mesenchymal stem cells derived exosomes had better survival, improved capillary density, reduced cardiac fibrosis, and restored long-term cardiac function (156, 157).

*Cardiac Progenitor Cells*

There are two types of cardiac progenitor cells (CPCs): embryonic CPCs and adult CPCs. Embryonic CPCs exist in the mammalian developmental heart, in common mesodermal lineage. Embryonic-like CPCs, named “developmental” CPCs, can be generated *in-vitro* from pluripotent stem cells. CPCs are defined by having self-renewing, clonogenic properties, and multipotent differentiation capabilities both *in-vitro *and *in-vivo *(158).

In contrast to the embryonic CPCs, adult CPCs, have been isolated from adult rodent and human hearts, although their role in potential function remains controversial. The cell-surface marker tyrosine kinase receptor *c-kit* has been routinely used to identify the adult CPCs (159). Cardiac c-kit^+^ cells isolated from the adult human heart and injected into the infarcted rodent myocardium have increased cardiac function. Recently, it was reported in the mouse models, all the c-kit^+^ cells were constitutively tagged, and thereby, the cardiac-derived c-kit^+^ cells localized in the injured heart could not be distinguished from the bone marrow-derived c-kit^+^ cells in the heart. Interestingly, the latest report has revealed that the majority (≈90%) of the resident c-kit^+^ cells in the rodent heart are endothelial cells, while cardiac c-kit^+^ (blood/endothelial lineage-negative) cells represent ≤ 10% of the total c-kit^+^ cells in the heart (160, 161). It is expected that the positive effects seen from the delivered c-kit^+^ cells in the post-MI heart could be due to the release of signaling molecules rather than the engrafted cells(162).

Apart from c-kit^+^, other progenitor-like cell populations have been identified as adult CPC-like cells, including Sca1^+^ cardiac cells, cardiosphere-derived cells, and cardiac side population cells. These cell types are heterogeneous in nature, and populations identified with different markers or approaches may be unique regarding molecular and physiological characteristics (163).

Cardiac progenitor cells (CPCs) obtained from the ventricle and right atrium contribute to cardiac myocytes and vascular endothelial cells turnover. These cells are similar to CSCs and can self-renewing, clonogenicity, and pluripotency (137). Matsuura *et al. *evaluated the efficacy of CPCs sheet in mice myocardial infarction model and found that CPCs differentiated into cardiomyocytes and led to an improvement of cardiac function (164). Another study in the MI rat model showed that extracellular vesicles of CPCs can enhance angiogenesis and cardiac function (165).

*Mesenchymal stem cells*

Mesenchymal stem cells** (**MSCs), multipotent cells with low immunogenicity, have been frequently used for heart cell therapy. However, experiments revealed that MSCs do not remain in target tissues, which can reduce their therapeutic administration. The most common sources for obtaining MSCs are bone marrow and adipose tissues (2). In a study, the efficacy of intramyocardial administration of allogeneic and autologous bone marrow-derived MSCs (BM-MSCs) has been performed in a clinical study that proved the enhancement of cardiac function in ischemic cardiomyopathy patients (166). Different *in-vitro *and *in-vivo* studies were done to find the best strategies to improve the efficacy of MSCs used via engraftment or engineered scaffold in myocardial ischemia (167, 168). An *in-vitro* study which used autologous BM-MSCs in a fibrin patch to enhance the efficacy of administrated MSCs showed that cardiac function improved by a significant increase in neovascularization (169). Kai *et al. *evaluated an ischemic cardiac function after applying MSCs seeded nanofibrous patch made of PCL/gelatin. This seeded patch improved angiogenesis and enhanced cardiac repair in the rat model of MI (170). In another study, cardiac function improvement was observed by applying a mixture of MSCs, thrombin, and fibrinogen to decellularized human heart tissue as an appropriate cell delivery vehicle (171). 

*Skeletal myoblast cells*

Skeletal myoblast cells are the first kind of cells applied for ischemic myocardial repair. Menasché reported that cardiac contractile function improved via patients’ intra-myocardial injection of autologous skeletal myoblasts (2, 172). Other clinical studies confirmed the effectiveness of autologous skeletal myoblasts via intra-myocardial transplantation of skeletal-derived cells (173, 174). However, the failure of myoblast cells to make a gap junction with host cells is yet a great concern. As myoblast cells do not express connexin-43, they cannot connect to other myocardial cells via gap junction, leading to an increase in ventricular arrhythmia risk (2).

**Cell sheets**

Despite the advantages of biodegradable scaffolds for tissue engineering, some concerns exist. Scaffolds as foreign materials may cause inflammation and infection in the body or stimulate the immune system. Furthermore, it is challenging to construct a cell dense tissue using biodegradable scaffolds. Decellularized tissues or cell sheets produced by cells as scaffolds are new options to solve these problems (175). Ishigami *et al. *used a cardiac tissue sheet with iPSCs in the infarcted myocardium site in mini pigs. Two weeks after infarction, they reported significant improvement in left ventricular function via patching the infarcted myocardium with human iPCs derived cardiac sheets (176). However, one of the concerns about cell sheet efficacy is the poor blood supply to the transplanted cell sheet. To overcome this problem, Kawamura *et al. *used the omental flap technique as a cell delivery system to enhance the efficacy of transplanted cell sheets into the site of infarcted myocardia. Significantly, increased ejection fraction was observed in the combination group in comparison to cell sheet only group, and tracking labeled human iPSCs derived cardiomyocytes with superparamagnetic iron oxide showed a greater survival rate in the group that used the omental flap technique (177).

**Direct reprogramming of cardiac fibroblasts**

Cardiac fibroblasts are key players in every stage of recovery from myocardial injury to fibrosis. Cardiac fibroblasts can be directly proliferated into cardiomyocytes (178). Cardiac fibroblasts are one type of most numerous cells in the heart. The iPSCs generated from Cardiac fibroblast, are more favorable for differentiation into cardiac cells, suggesting that they could be a source of cardiomyocytes for cell therapy and heart tissue engineering (179). Although evidence has shown that cardiac fibroblasts contribute to regeneration after injury, the complexity of their function remains unknown. It is also interesting whether cardiac reprogramming will affect these cells to synergetic benefit for heart tissue repair (180). Expression of some transcription factors such as Gata4, Mef2c, Tbx5, ESRRG, MESP1, ZFPM2, and Myocardin can reprogram cardiac fibroblasts into CM-like cells. However, few studies report that different combinations of transcription factors could generate cardiac cells from fibroblasts, but they failed to beat (181, 182). Direct cardiac reprogramming induces cardiomyocytes *in-situ* from cardiac fibroblasts. This could be a new therapeutic method for heart regeneration without cell transplantation. It is necessary and important to expand our understanding of cardiac fibroblasts about their characteristics, behaviors, and functions in humans. Further advances in cardiac reprogramming research are necessary for cardiac regenerative therapy in humans. 

**Heart on a chip**

A heart-on-chip is a microfluidic chip that mimics the mechanisms of a heart to test medicine and observe the reaction of heart cells. Animal models can help understand biological and physiological processes but often fail to exactly represent human cardiotoxicity due to inter-species differences (183). The inaccurate results can be dangerous, especially for heart medication doses. There can also be substantial differences among humans due to age, race, or genetic diversity. A heart-on-chip is a simple innovative way to reproduce cardiac tissue in three dimensions. The idea behind this microphysiological device is to find a simple and cheap method to apply for the study of cardiac diseases, cardiac drug development, and cardiotoxicity testing, personalized medicine, and damaged heart tissue regeneration. To create a valuable model, the microchip should mimic the heart’s main properties: mechanical contractions, molecular transport, electrical activity, and specific responses to some drug stimulation. It is interesting to add specific measurement systems, for potential recordings of contraction or action, or even calculation of tissue elastic fibers (184). The heart-on-chip should reproduce the cellular organization level in a living heart by exhibiting sarcomeres assembling with aligned tissue structure. The heart is also one of the rare organs with active tissues that show intrinsic contractions. Moreover, the tissues are known to have a better cardiac differentiation when stimulated by a stretching pulse (185). The contraction movement is therefore extremely important for a robust model with mature cardiomyocytes. The heart is a pump but also a muscle, which means energy consumption and molecular transport. Another key point to build a future automatic large-scale method for the pharmaceutical industry is to obtain measurements data directly from the microchip to evaluate the potentiality of the tested treatment. Professor Wikswo and his team built an engineered 3D cardiac tissue: the I-Wire Heart-on-Chip incorporating wires that can be used for both electrical stimulation and chronic measures (186). 

Heart-on-chip technology has potential for pharmacologic and research applications, especially when using integrated registration systems for contraction and action potential. Having a reliable heart-on-chip opens the door for a human-on-chip: a multi-organ microdevice. It is essential for drug testing to assess not only the cardiotoxicity but also the global inter-organs interactions.

**Definition of ideal cell type**

The ideal cell for myocardial regeneration should be easy to obtain in sufficient number, capable of proliferation and differentiation into functional cardiomyocytes that couple to neighboring cells, and applicable without the need for an immunosuppressant. None of the mentioned cell types meets all these criteria. The goal of myocardial regeneration is to replace the dead myocardium with enough contractile cells that beat synchronously with the host cardiomyocytes to restore cardiac function. The cell source for myocardial regeneration should generate cells that fulfill at least two prerequisites, generating active force and electromechanical coupling with host cardiomyocytes. As discussed above, neither skeletal myoblasts nor BM-derived cells, the two cell types currently used in clinical trials, meet these two criteria. The modest improvement in cardiac function observed in clinical trials cannot be attributed to these cells’ significant formation of functional cardiomyocytes. In contrast, CSCs and hESCs can truly differentiate into cardiomyocytes that couple with surrounding cardiac cells both *in-vitro* and *in-vivo* (187). Although several hurdles need to be overcome before they can be applied in clinical practice, CSCs and hESCs are possibly the best candidates for real myocardial regeneration (188).

**Pharmacologic agents in cardiac tissue engineering**

**Figure 3:**
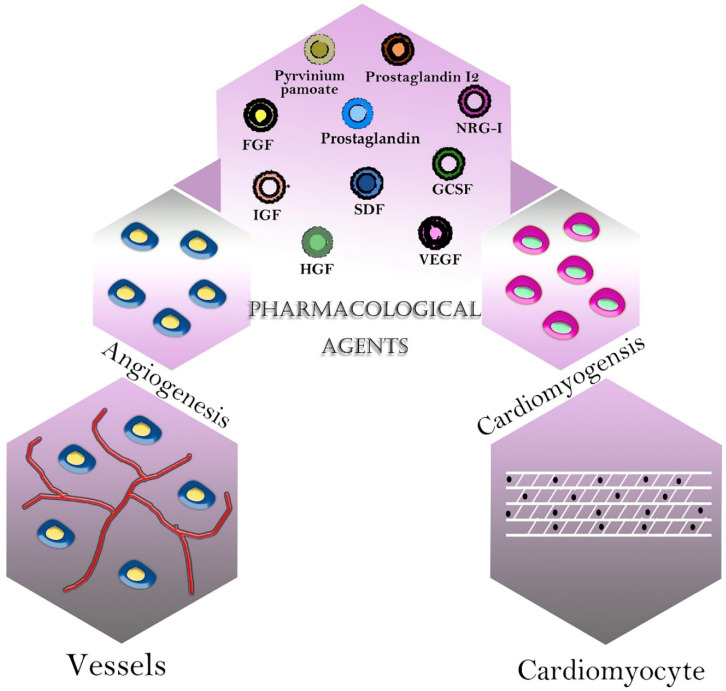
Pharmacological agents in cardiac tissue engineering

Many small-molecule drugs, growth factors, and other active agents have been used for cardiac tissue engineering (Figure 3). These drugs are used to promote the growth of tissue or modulate the inflammatory response (189). Degradation products of these natural and synthetic macromolecules have anti-inflammatory properties (Table 3). 

Some anti-inflammatory drugs such as paclitaxel and sirolimus, commonly used for drug-eluting stents (190) and post balloon angioplasty (191-195), are not useful for cardiac tissue engineering approaches because they also prevent cell proliferation and tissue formation. Tissue formation is necessary for the early phase of tissue engineering, but later new tissue deposition should decrease and the cells should become more similar to native tissue. Some strategies have used doxycycline to reduce matrix metalloproteinase expression and preserve matrix structure. However, doxycycline inhibits endothelial cell proliferation, which will prevent the formation of a functional endothelium (196). In this case, careful control over the dose and timing of the anti-inflammatory agents is required if these agents incorporate within a tissue engineering strategy.

The molecular mechanisms of cardiomyogenic differentiation of stem cells are unclear, and current protocols for *in-vitro *differentiation of ESCs into cardiomyocytes by growth factors are inefficient and non-selective. To make stem cells a relevant source for clinical cell therapy, methods need to be developed to enable large-scale, feeder-independent production of cardiac progenies free of contamination by animal products from the culturing process. Therefore, pharmacological agents that induce directed differentiation of stem cells to produce a large number of homogeneous populations of cardiac-committed cells undoubtedly bear the potential to facilitate the application of stem cells for myocardial regeneration. Several growth factors and small molecules such as retinoic acid and dimethyl sulfoxide, enhance the formation of beating cardiac myocytes in a mouse ESC-embryoid body cell differentiation system (136). More recently, cell-based high-throughput screening has been used to efficiently identify small molecules that induce cardiac differentiation of ESCs in the absence of embryoid body formation. Using ESCs stably transfected with cardiac-specific α-myosin heavy chain (α-MHC) promoter-driven enhanced green fluorescent protein as a reporter, Takahasji *et al. *screened 880 compounds approved for human use and found that ascorbic acid enhances differentiation of ESCs into cardiac myocytes(197). This effect of ascorbic acid is not related to its antioxidant property because other antioxidants do not have any such effect. Wu *et al. *identified a series of diaminopyrimidine compounds called cardiogenol A-D that efficiently and selectively induce mouse ESCs to differentiate into cardiomyocytes(198). 

Meanwhile, some attempts to identify molecules to enhance the formation of cardiomyocytes by BM-derived stem cells or CSCs have also been made in conventional cell culture experiments. For example, it has been reported that 5-azacytidine promotes differentiation of CSCs and MSCs into cardiomyocytes and that the nonapeptide oxytocin enhances differentiation of CSCs to beating cardiomyocytes. Besides differentiation enhancement, the proliferation of CSCs, either *ex-vivo *or *in-vivo*, could facilitate the application of CSCs in myocardial regeneration. Non-toxic compounds capable of enhancing proliferation and cardiac differentiation of these stem cells hold great promise. Subsequent analysis of changes in intracellular signaling molecules induced by these compounds could identify key signal pathways involved in cell proliferation and cardiomyogenic differentiation, enabling drug design (199).

*Small molecules*

Small molecule drugs represent a promising treatment for MI. These compounds are inexpensive. Advances in chemical synthesis result in the production of large amounts of different molecules. A small-molecule structure enables modification to optimize specificity, stability, and efficacy. Small molecule drugs are currently at an early stage of development for myocardial regeneration (200). An example of a small molecule, Dipeptidylpeptidase IV (DPP-IV) is a membrane-bound peptidase that cleaves Stromal cell-derived factor-1 (SDF-1). Pharmacological inhibition of DPP-IV stabilizes myocardial SDF-1 after MI, thereby recruiting CXCR4 + circulating stem cells to regenerate the heart. Zaruba *et al. *administered Diprotin A, a small molecule with DPP-IV inhibitor, G-CSF, to mobilize stem cells or a combination of both in a mouse MI model. Combining G-CSF and DPP-IV inhibition resulted in an increase in CXCR4 + cell homing to the myocardium, neovascularization, increased myocardial function, and survival. Only the combination of Diprotin A and G-CSF treatment significantly increased myocardial function, ing multimodal therapeutic strategies’ potential. A Phase III clinical trial with a DPP-IV inhibitor, Sitagliptin, combined with G-CSF in patients with acute MI demonstrated well tolerance, but the efficacy has not been published yet (201).

*Prostaglandins*

Prostaglandins are endogenous small-molecule that derivate from fatty acid and mediate a variety of physiological effects. Prostaglandin E2 (PGE2) and prostaglandin I2 (PGI2) have regenerative effects in the injured myocardium and therapeutic potential.

Intraperitoneal administration of PGE2 induced cardiomyocyte regeneration at the infarct area in a murine model of MI, whereas prostaglandin I2 (PGI2) did not have these effects. PGE2 increased the regulatory effect for cardiomyogenic differentiation of cells. Besides, PGE2 treatment rescued the ability of old mouse hearts to regenerate cardiomyocytes, especially in the infarct zone. However, PGE2 is rapidly metabolized *in-vivo*, and so repeated administration is necessary. This situation supports the need for long-term release products within encapsulation or synthesis of more stable prostaglandins (202).

PGI2 is a vasodilator and potent anti-coagulant for the treatment of hypertension (203). Like PGE2, PGI2 has a short half-life *in-vivo*. Delivery of ONO1301, a stable small molecule PGI2 agonist on an epicardial collagen patch in a model of dilated cardiomyopathy, demonstrated expression of Hepatocyte Growth Factor (HGF), Vascular endothelial growth factor (VEGF), Stromal cell-derived factor-1 (SDF-1), and G-CSF. ONO1301 treatment increased myocardial vascularization, reduced fibrosis, and prolonged survival. In another study, ONO1301 loaded PLGA/microspheres were injected intramyocardially in a mouse model of acute MI and increased local HGF and VEGF expression, increased vascularisation of the infarct border zone by day 7, decreased left ventricular dilatation, and improved survival by day 28. In a phase I clinical trial, ONO1301 was administered orally, but the study was discontinued because of diarrhea in participants. These results revealed that it should be used locally, and off-target effects should be controlled.

*Pyrvinium pamoate*

Pyrvinium Pamoate (PP) is an FDA-approved anthelmintic drug, which inhibits NADH-fumarate reductase activity essential for the anaerobic respiration of parasitic worms. Murakoshi *et al. *demonstrated that the administration of PP could produce a cytotoxic effect in fibroblasts which proliferate in the myocardial scar after infarction and enable anti-fibrotic therapy. PP was administered orally, daily, one day after MI. There was a significant reduction in the presence of fibroblasts in the infarct and border zone at the end of the study, and left ventricular ejection fraction (LVEF) increased in PP treated animals. Also, vascularization has been shown(204).

In another study, researchers administered an intramyocardial injection of PP at the time of coronary artery ligation in a mouse model of MI and observed a significant increase in animal mortality upon PP treatment. It is due to early-stage administration of PP in which cardiomyocyte death occurred and highlights the importance of administration time. Surviving animals did not display a significant cardiac regeneration or reduction of fibrosis. This revealed that an injection of PP may not produce significant myocardial retention up to the time fibrosis. The drug delivery at the time of initiation of fibrosis may improve treatment outcomes (205). Because of the cytotoxicity of this drug, the use of targeted nanoparticulate carriers is necessary to ensure increased specificity for fibroblasts and decreased toxicity risk (205).

*Growth factors and proteins*

Among the different therapeutic agents for heart regeneration, peptides and proteins represent well-defined sources. The increased accessibility to these therapeutics and the advances in chemical modifications to enhance *in-vivo* protein half-life and minimize immunogenicity (206) offer a broad range of new therapeutic agents. Modified peptides and proteins can promote cardiac regeneration by activating endogenous cardiac progenitor cells, cardiomyocyte proliferation, and the recruitment of progenitor cells to damaged myocardium. 

In the light of angiogenesis, VEGF has been demonstrated to be a major regulator of vascularization under hypoxic conditions. VEGF administration after MI can induce angiogenesis and improve cardiac function. Despite its efficacy in preclinical models, VEGF did not successfully translate to clinical applications due to the risk of nitric oxide-mediated hypotension(207). Additionally, some concerns about the progression of metastatic tumor lesions as side effects of the prolonged administration of angiogenic growth factors have been raised (208).

SDF-I has been described as a potent stem cell homing agent involved in the regeneration of the vasculature (209). By binding to the CXCR4 receptor, SDF-I increases the recruitment of endothelial progenitor cells (206). This mechanism suggests a safe application in the induction of angiogenesis. But, a major drawback of using SDF-I is rapid degradation by DPP-IV and matrix metalloproteinases in the heart. To compensate for this disadvantage and improve its pharmacokinetics and activity, nanofiber-mediated delivery of SDF-I has been suggested (210). 

Neuregulin 1 (NRG-I), a member of the epidermal growth factor family increase proliferation of cardiomyocytes through ErbB4 receptor binding. Patients with stable chronic heart failure showed an improved cardiac function after daily injections of NRG-I for eleven days (211).

HGF is a mesenchyme-derived pleiotropic factor that stimulates the proliferation of hepatocytes. The antiapoptotic effect of HGF on cardiomyocytes has been demonstrated in rats after myocardial ischemia and reperfusion injury. Also, HGF promotes angiogenesis and progenitor cell recruitment (212). 

The growth and differentiation of stem cells may be supported by Insulin-like growth factor 1 (IGF-I). This hormone binds a tyrosine kinase receptor and enhances cell survival. IGF-I has been shown to reduce myocardial necrosis and apoptosis (213). However, higher doses have been associated with hypotension and tachycardia. To increase the bioavailability and control the release of growth factors in the cardiac tissue, drug delivery systems have been suggested as a means to protect and accumulate the protein cargo (214). 

The feasibility of controlled delivery using polymeric carriers was also shown for other proteins involved in the repair of the damaged heart (215). 

G-CSF induces the proliferation of hematopoietic stem cells with the capacity to regenerate the infarcted myocardium, an effect which was observed only in patients who received G-CSF early after MI (216). 

Increasing knowledge of the various molecules involved in cardiac tissue engineering and new developments in pharmaceutical drug combinations to maximize therapeutic potential is expected to occur soon. Also, the formulation of these therapeutic agents in drug delivery systems will provide a safer and more efficient administration, leading to improve clinical outcomes.

**Table 1:** Comparison of natural and synthetic scaffolds properties (217).

**Characteristic**	**Natural scaffolds**	**Synthetic scaffolds**
Immunogenicity(218)	Antigens could be removed	Unknown depends on the materials
Reproducibility(219)	High variability between donor scaffolds	High possibility of control
Differentiation(220)	Maintain native integrin-binding sites	Lack specific integrin-binding site
Biocompatibility(221)	High variability depending on the source of the material	Poor compatibility
Biodegradability(222)	Undefined degradation rate	Poor; potential toxic degradation materials

**Table 2:** Various types of cells used for cardiac tissue regeneration (223).

**Cell types**	**Origin**	**Advantages**	**Disadvantages**
Mesenchymal stem cells(224)	Bone marrow, adipose tissue, umbilical cord	Antiapoptotic, vasculogenic, lesser side effects, no immunogenicity, easily available.	Low cell count, weak electrical and myogenic properties, less regenerative abilities, and negative effects on cardiac function
Cardiac stem cells and cardiac progenitor cells(225)	Heart	High regeneration ability,antiapoptotic, angiogenic, no immunogenicity	Weak myogenic properties, low cell count, age‐dependent response, limited growth
Embryonic stem cells (226)	Embryo	Efficient myogenic	Teratoma, ethical concerns, incontrollable differentiation, immunogenicity, arrhythmias
Skeletal myoblast cells(227)	Skeletal muscles	Myogenic, minimal side effects	Contamination with other cells, low vasculogenic
Induced pluripotent stem cells(228)	Skin	Differentiate into a variety of cell types, high cell count, disease modeling	Arrhythmia, genetic instability, tumorigenicity, immunogenicity, requirement of vector system, time, and cost

**Table 3:** Various active agents for cardiac tissue regeneration (229).

**Active agent**	**Type**	**Effect**
Prostaglandin E2 (PGE2) (230)	Endogenous small-molecule fatty acid derivative	Cardiomyocyte replenishment
Prostaglandin I2 (PGI2) (231)	Vasodilator and potent anti-coagulant	Expression of cardioprotective HGF, VEGF, SDF-1, and G-CSF
Pyrvinium Pamoate (232)	Anthelmintic drug	Anti-fibrotic
Dipeptidylpeptidase IV (DPP-IV) inhibition (233)	Membrane-bound peptidase	Increasing CXCR4 + circulating stem cells and regeneration
Vascular endothelial growth factor (VEGF) (234)	Signaling protein	Regulator of vascularization under hypoxic conditions
stromal cell-derived factor 1 (SDF-I) (235)	Potent stem cell homing agent and vascularization effect	Recruitment of endothelial progenitor cells
Neuregulin 1 (NRG-I) (236)	Epidermal growth factor	Increasing cell cycle activity and proliferation through ErbB4 receptor binding
Insulin-like growth factor 1 (IGF-I) (237)	Hormone	Enhancement of cell survival through tyrosine kinase receptor binding, reduction of myocardial necrosis and apoptosis
Granulocyte colony-stimulating factor (G-CSF) (238)	Cytokine and hormone	Proliferation of hematopoietic stem cells and regeneration

## Conclusion

The ability to construct engineered heart tissue or regenerate damaged myocardial tissue following aging, disease, or genetic abnormality is a great promise for researchers. However, myocardial tissue engineering still has significant difficulties and challenges. One of these difficulties is the design of bioactive scaffolds, which allow contractility and conductivity. Also, there is a need for the development of strategies to create vasculature in engineered myocardial tissue. Biomaterials are widely used in tissue engineering and regenerative medicine because of their biodegradability and biocompatibility characteristics. One of the main characteristics of biomaterials is their ability to incorporate various growth factors and cytokines and control their release. Thus, biomaterials can serve as a controlled and sustained delivery of growth factors and cytokines to ameliorate inflammation, improve angiogenesis, reduce fibrosis, and generate functional cardiac tissue. Moreover, they can address some of the challenges of stem cell therapy. Also, they can improve stem cell survival and retention, enhance the delivery of the factors produced by the cells, support differentiation, and boost their therapeutic efficacy. All these benefits besides cell selection can promote the development of cardiac function after failure. 

Furthermore, the best source of cells and optimal doses of various growth factors and cytokines should be considered to create functional cardiac tissue and improve heart function. Other important goals are to increase our understanding of tissue formation, function, and failure. In addition to *in-vitro *myocardial construction, more research should be carried out for regenerating myocardium *in-situ*. There is much evidence to explain that the heart should be capable of self-regeneration. Future studies are needed to study the potential of other agents in myocardial regeneration. Successful strategies will solve the problem of organ donor shortage and can be used for the repair of the injured myocardium. The ability to provide some way for repairing damaged myocardium will be a gold impact on science. However, the success of this concept depends on carefully selecting cells, materials, and other agents to achieve this object.

## Conflict of interests

The authors declare there is no conflict of interests.
